# An Abnormal Host/Microbiomes Signature of Plasma-Derived Extracellular Vesicles Is Associated to Polycythemia Vera

**DOI:** 10.3389/fonc.2021.715217

**Published:** 2021-11-25

**Authors:** Monica Barone, Martina Barone, Francesca Ricci, Giuseppe Auteri, Giulia Corradi, Francesco Fabbri, Valentina Papa, Erika Bandini, Giovanna Cenacchi, Pier Luigi Tazzari, Nicola Vianelli, Silvia Turroni, Michele Cavo, Francesca Palandri, Marco Candela, Lucia Catani

**Affiliations:** ^1^ Department of Medical and Surgical Sciences, University of Bologna, Bologna, Italy; ^2^ Department of Pharmacy and Biotechnology, University of Bologna, Bologna, Italy; ^3^ Istituto di Ricovero e Cura a Carattere Scientifico (IRCCS) Azienda Ospedaliero-Universitaria di Bologna, Istituto di Ematologia “Seràgnoli”, Dipartimento di Medicina Specialistica, Diagnostica e Sperimentale, Università di Bologna, Bologna, Italy; ^4^ Servizio di Immunoematologia e Trasfusionale, Istituto di Ricovero e Cura a Carattere Scientifico (IRCCS) Azienda Ospedaliero-Universitaria di Bologna, Bologna, Italy; ^5^ Biosciences Laboratory, Istituto di Ricovero e Cura a Carattere Scientifico (IRCCS) Istituto Romagnolo per lo Studio dei Tumori (IRST) "Dino Amadori", Meldola, Italy; ^6^ Department of Biomedical and Neuromotor Sciences, University of Bologna, Bologna, Italy; ^7^ Istituto di Ematologia “Seràgnoli”, Istituto di Ricovero e Cura a Carattere Scientifico (IRCCS) Azienda Ospedaliero-Universitaria di Bologna, Bologna, Italy

**Keywords:** polycythemia vera, cancer, extracellular vesicles, microbial DNA cargo, gut microbiota

## Abstract

Polycythemia Vera (PV) is a myeloproliferative neoplasm with increased risk of thrombosis and progression to myelofibrosis. Chronic inflammation is commonly observed in myeloproliferative neoplasms including PV. The inflammatory network includes the extracellular vesicles (EVs), which play a role in cell-cell communication. Recent evidence points to circulating microbial components/microbes as potential players in hemopoiesis regulation. To address the role of EVs in PV, here we investigated phenotype and microbial DNA cargo of circulating EVs through multidimensional analysis. Peripheral blood and feces were collected from PV patients (n=38) and healthy donors (n=30). Circulating megakaryocyte (MK)- and platelet (PLT)-derived EVs were analyzed by flow cytometry. After microbial DNA extraction from feces and isolated EVs, the 16S rDNA V3-V4 region was sequenced. We found that the proportion of circulating MK-derived EVs was significantly decreased in PV patients as compared with the healthy donors. By contrast, the proportion of the PLT-derived EVs was increased. Interestingly, PV was also associated with a microbial DNA signature of the isolated EVs with higher diversity and distinct microbial composition than the healthy counterparts. Of note, increased proportion of isolated lipopolysaccharide-associated EVs has been demonstrated in PV patients. Conversely, the gut microbiome profile failed to identify a distinct layout between PV patients and healthy donors. In conclusion, PV is associated with circulating EVs harbouring abnormal phenotype and dysbiosis signature with a potential role in the (inflammatory) pathogenesis of the disease.

## Introduction

Polycythemia Vera (PV) is a chronic myeloproliferative neoplasm (MPN) characterized by clonal expansion of the erythrocyte mass. There is often concurrent stimulation of myeloid and megakaryocytic lineages leading to increased white blood cell and platelet production, respectively. Molecular pathogenesis relies on constitutive activation of the JAK-STAT pathway that is responsible for abnormal myeloproliferation and increased production of circulating inflammatory cytokines (1, 2). Clinical phenotype includes increased risk of thrombosis and progression toward myelofibrosis and/or leukemia. PV management is primarily focused on minimizing the thrombotic risk, representing the main cause of morbidity and mortality (3, 4). All MPNs are associated with an important inflammatory response as demonstrated by the presence of high plasma levels of inflammatory cytokines as well as constitutional symptoms alleviated by anti-inflammatory therapies. These inflammatory cytokines are synthetized by the mutated and non-mutated hematopoietic cells as well as by non-hematopoietic cells such as mesenchymal stromal cells (5–8). Inflammation is an important thrombotic risk factor in PV (3, 4, 9).

Over the past few years, extracellular vesicles (EVs) have emerged as key modulators of immunity and inflammation (10). EVs from both immune and nonimmune cells, such as mesenchymal stem cells and endothelial cells, contribute to antigen-specific and nonspecific immune regulation. They also likely play a role in modulating inflammatory and autoimmune diseases (11). EVs are membrane-surrounded particles that are released by a broad variety of cells, either eukaryotic or prokaryotic, with effects on cell signaling. EV cargos are enriched in nucleic acids, proteins, and lipids and these bioactive molecules can be delivered to recipient cells to influence their biological properties and modify surrounding microenvironment or distant targets. Most circulating EVs are of megakaryocyte (MK-EVs) and platelet (PLT-EVs) origin. Recently, the number as well as the cargo, including proteins, microRNA and long non-coding RNA, have been reported to be upregulated in the EVs of patients with blood cancers, suggesting that circulating EVs might be a diagnostic marker for these disorders (12, 13).

Interestingly, evidence indicative of the presence of a microbial component in the blood of healthy human individuals is steadily accumulating (14, 15). Furthermore, EVs may contain a range of immunostimulatory microbe-associated molecules, including Gram-negative bacteria derived-lipopolysaccharide (LPS), whose circulating levels have been reported to be increased in patients with intestinal barrier dysfunction (16, 17). Of note, it has been hypothesized that peculiar cargo of microbiomes components in EVs may control for their overall inflammatory potential.

Based on this, here we hypothesized that the combined evaluation of circulating EVs and the carried microbiomes components could provide a signature for PV patients and possibly contribute to add further knowledge of the pathogenesis of the disease.

## Methods

The “EV-PV” study is a monocenter clinical-biological study promoted by the Institute of Hematology “L. and A. Seràgnoli”, S. Orsola-Malpighi Bologna University Hospital, and performed in collaboration with the Department of Pharmacy and Biotechnology, University of Bologna. After approval by the local Ethics Committee, the EV-PV study was conducted according to the Helsinki declaration. Informed consent was obtained from all subjects.

### Collection of Peripheral Blood and Platelet Poor Plasma Preparation

Briefly, EDTA-anticoagulated peripheral blood and fecal samples were collected from PV patients, regardless of the time of diagnosis, and from age- and sex-matched healthy donors (HD). PV was diagnosed according to the 2016 WHO classification (18). After discarding the first 2 ml of blood, Platelet Poor Plasma (PPP) was obtained (within 2 hours from blood collection) after two consecutive centrifugations at 2500 × g for 15 min at room temperature. PPP was then aliquoted and stored at -80°C until testing.

### Identification and Characterization of Circulating EVs by Flow Cytometry

Circulating MK (CD61+/CD62P-)- and PLT (CD61+/CD62P+)-EVs were analyzed by flow cytometry (Navios, Beckman Coulter, Milan, Italy) in PPP, after thawing at 37°C. PPP (100 µl) was incubated at 4°C for 15 min with antibodies, then diluted 1:3 and acquired immediately (List of monoclonal antibodies according to EVs subtype is shown in **Table S2**). To detect EVs, the instrument was calibrated with MegaMix Beads (Stagò, Marseille, France). Fluorescence gated polystyrene beads of different sizes were used to determine the gates identifying big (500-900 nm), small (200-300 nm) and nano (100-160 nm) EVs, as previously described (19). The Violet Side Scatter laser (VSSC) was used as a trigger signal to discriminate noise. Our analysis was focused on big EVs that were identified by using the size and ability to bind specific monoclonal antibodies. Matched isotype controls were used to select the cut-off. By using the defined gate for big EVs, all events positive for marker staining were recorded and expressed as a percentage of positive EVs.

### Circulating EV Isolation, Enumeration, Morphology, and Phenotype Characterization

EVs were isolated from thawed PPP (2 ml at 37°C) by ultracentrifugation at 100,000 × g for 2 hours at 4°C with Optima L-90 K ultracentrifuge (Beckman Coulter) equipped with Type 50.2 Ti rotor, as previously described (20). After centrifugation, pelleted EVs were resuspended and washed with twice filtered (filter pore size, 0.22 µm) Dulbecco’s PBS (DPBS; Sigma Aldrich). Finally, EVs were resuspended in saline buffer solution with 1% DMSO and stored at − 80°C until use. EV enumeration and purity were assessed by using the NanoSight technology (NanoSight NS300-Malvern Panalytical Ltd., Royston, United Kingdom) and nanoparticle tracking analysis software (NTA Proprietary Software-Malvern Panalytical Ltd.). Transmission electron microscopy analysis of the isolated EVs was also performed. Samples were processed for negative staining by adsorption, washing and staining steps. The suspension (10 µl) was placed on a carbon-coated grid and after 30 sec of adsorption, it was slowly and gently removed by bibulous pieces of paper without touching the grid directly. Then, a series of droplets of distilled water were used to remove interfering salts. Ten microliters of water 1% uranyl acetate solution, pH 4.4 were used for negative staining, 5-10 sec. Stain droplets were gently removed as described above. After drying, grids were observed by a Philips CM 100 (TSS microscopy, Hillsboro, OR, USA), recorded by digital camera (Olympus, Milan, Italy), and digitally measured by iTem software. Isolated EVs were characterized by cytofluorimetric analysis using monoclonal antibodies against tetraspanins (CD9, CD63, CD81), CD61, CD62P, and lipopolysaccharide (LPS) (List of monoclonal antibodies according to EVs subtype is shown in **Table S2**). Conjugated mouse isotypic IgG was used as a control. Briefly, EVs (2×108) were stained at 4°C for 15 min, then diluted 1:3 and acquired immediately, as above described. Tetraspanins analysis was focused on total isolated EVs while the other markers were analyzed on big EVs.

### Microbial DNA Extraction From Feces and Isolated EVs

Microbial DNA was extracted from feces (250 mg) and isolated EVs (2ml of PPP) using the repeated bead-beating plus columns method, as previously described with a few modifications (21). In brief, all samples were suspended in 1 ml of lysis buffer (500 mM NaCl, 50 mM Tris-HCl pH 8, 50 mM EDTA, and 4% (w/v) SDS), added with four 3-mm glass beads and 0.5 g of 0.1-mm zirconia beads (BioSpec Products, Bartlesville, OK) and bead-beaten in a FastPrep instrument (MP Biomedicals, Irvine, CA) at 5.5 movements/sec for 1 min. Only one homogenization step was performed for EVs samples, while for stool samples it was repeated three times, incubating the samples on ice for 5 min between treatments. After incubation at 95°C for 15 min, all samples were centrifuged at 13,000 rpm for 5 min. Nucleic acids were precipitated by adding 260 µl of 10 M ammonium acetate and one volume of isopropanol. The pellets were then washed with 70% ethanol and suspended in 100 µl of TE buffer (10 mM Tris-HCl, 1 mM EDTA, pH 8). RNA was removed by treatment with 2 µl of 10 mg/ml DNase-free RNase at 37°C for 15 min. Protein removal and column-based DNA purification were performed by using the DNeasy Blood and Tissue kit (QIAGEN, Hilden, Germany) and following the manufacturer’s instructions. Template-free controls (i.e. RPMI medium and extraction kit reagents) were processed as well, in the same way as the samples. DNA was quantified with the NanoDrop ND-10000 spectrophotometer (NanoDrop Technologies, Wilmington, DE).

### 16S rRNA Gene Amplification and Sequencing

The V3-V4 hypervariable region of the 16S rRNA gene was amplified from DNA extracted from isolated EVs, stool samples and no-template controls, by using the 341F and 785R primers with Illumina overhang adapter sequences, as previously described by Klindworth et al. (22). PCR reactions were performed by using KAPA HiFi HotStart ReadyMix (Roche, Mannheim, Germany), in a Thermal Cycler T (Biometra, Göttingen, Germany) with the following gradient: 3 min at 95°C for the initial denaturation, 25 cycles of denaturation at 95°C for 30 sec, annealing at 55°C for 30 sec, and elongation at 72°C for 30 sec, and a final elongation step at 72°C for 5 min. PCR products of around 460 bp were purified using a magnetic bead-based system (Agencourt AMPure XP, Beckman Coulter), and a limited-cycle PCR using Nextera Technology was performed to obtain the indexed library, followed by a second clean-up step as described above. Indexed libraries were pooled at equimolar concentration of 4 nM, denatured with NaOH 0.2 N, and diluted to 5 pM before loading onto the Illumina MiSeq flow cell. The 2 × 250 bp paired-end sequencing protocol was performed according to the manufacturer’s instructions (Illumina, San Diego, CA). Sequencing reads were deposited in the National Center for Biotechnology Information Sequence Read Archive (NCBI SRA) under the following project number: PNRxxx.

### Bioinformatics and Biostatistics

Statistical analysis was performed at the Department of Pharmacy and Biotechnology and at the biostatistics laboratory of the MPN Unit at the Institute of Hematology “L. and A. Seràgnoli”, IRCCS Azienda Ospedaliero-Universitaria di Bologna.

Raw sequences were processed using a pipeline that combines PANDAseq (23) and QIIME 2 (24). After length (min/max = 350/500 bp) and quality filtering (default parameters), cleaned reads were binned into amplicon sequence variants (ASVs) using DADA2 (25, 26). Taxonomy was assigned through the VSEARCH algorithm (27), using the Greengenes database as a reference (release May 2013). All singleton ASVs were discarded. Moreover, for EVs, ASVs were considered putative contaminants and therefore removed when their mean relative abundance did not exceed 20% of that in controls, similarly to what was previously applied by Dash and colleagues (28). Alpha diversity was evaluated using two different metrics: Shannon and inverse Simpson (1/D). The Jaccard similarity index was used to construct Principal Coordinates Analysis (PCoA) plots.

Statistical analyses were performed using R 3.6.1, using R Studio 1.2.1335 and the packages vegan (29), made4 (30) and stats (31). The significance of data separation in the PCoA was tested by means of a permutation test with pseudo-F ratio (function adonis of the vegan package). Wilcoxon rank-sum test was used to assess significant differences between groups (for intra- and inter-individual diversity as well as taxon relative abundance), while Kruskal-Wallis test was used for multiple comparisons. P values were corrected for multiple testing using the Benjamini-Hochberg method, and a false discovery rate (FDR) ≤0.05 was considered statistically significant.

As for the phenotype of EVs, statistical analysis was performed with GraphPad (GraphPad Software Inc., La Jolla, CA). The differences between the groups were analyzed with Mann-Whitney, Chi-square or Spearman’s correlation tests, as appropriate. P values were considered significant when ≤0.05 (2-tailed).

## Results

### Study Cohort

Thirty-eight PV patients (35 JAK2V617F and 3 JAK2Exon12-mutated) were included into the study after a median time from PV diagnosis of 2.6 years (range, 0.1-13.6). At the time of enrollment, all patients had received phlebotomies and antiplatelet therapy (mainly low-dose aspirin); hydroxyurea was ongoing in 81.6% of patients. No patients had received therapy with anagrelide, busulfan, interferons or ruxolitinib. Peripheral blood/fecal samples were also collected in 30 HD. Compared to PV patients, HD presented significantly lower platelet/leukocytes/hematocrit levels and were less frequently treated with low-dose aspirin (**Table S1**).

### Characterization of PLT-EVs, MK-EVs, and LPS-EVs

**Figure 1:**
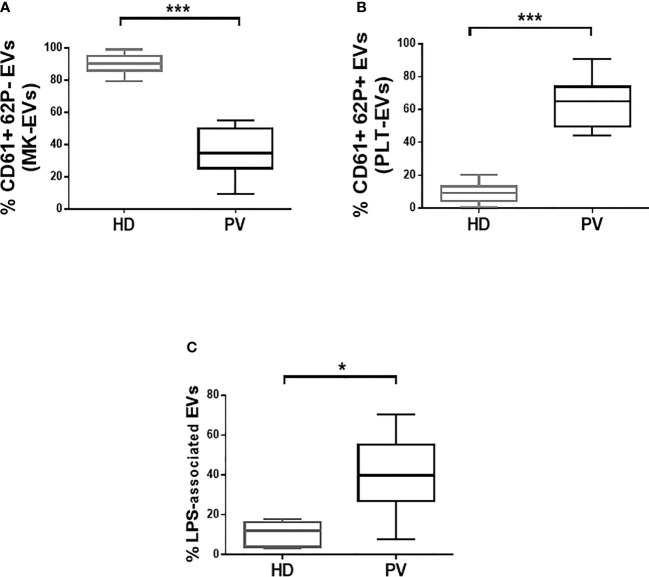
Proportion of plasma MK- and PLT-EVs, and of isolated LPS-associated EVs in patients with PV and HD. **(A, B)** MK-EVs and PLT-EVs profile of PV patients (n = 38) and HD (n = 30). Data are expressed as percentage of MK-EVs and PLT-EVs and presented as min to max with median (Mann-Whitney test; *p < 0.05; ***p < 0.001). **(C)** Proportion of isolated LPS-associated EVs of PV patients (n = 28) and HD (n = 20). Data are expressed as percentage and presented as min to max with median (Mann-Whitney test; *p < 0.05).

The profile of PLT-EVs and MK-EVs was assessed by flow cytometry in the plasma of PV patients and HD (**Figure S1A**). Plasma MK-EVs were significantly decreased in PV patients (p<0.001; **Figure 1A**); by contrast, PLT-EVs were significantly increased (p<0.001; **Figure 1B**). Both in PV and HD, the proportion of plasma MK-EVs and PLT-EVs was not influenced by sex or age >60 years. No differences were also observed between PV patients with/without previous thrombosis or HU treated/untreated.

We also analyzed the phenotype, size, and morphology of the isolated EVs. Interestingly, compared to HD, PV patients showed increased proportion of the isolated LPS-associated EVs (p<0.05; **Figure 1C** and **Figure S1B**). The tetraspanins (CD81, CD63 and CD9) were highly expressed by the majority of the isolated EVs from both patients and HD (**Figures S2A**–**D**). Consistent with the circulating EVs data, the proportion of isolated MK-EVs and PLT-EVs of PV patients was decreased and increased, respectively, compared to those of HD (p<0.01; **Figures S2E, F**). At variance with the concentration (**Figure S3A**), the isolated EVs of PV patients were significantly smaller compared to those of the HD counterpart (p<0.001; **Figures S3B, C**). Transmission electron microscopy analysis showed round-shaped EVs in both PV patients and HD (**Figure S3D**).

### Characterization of EV-Associated Microbial DNA

**Figure 2:**
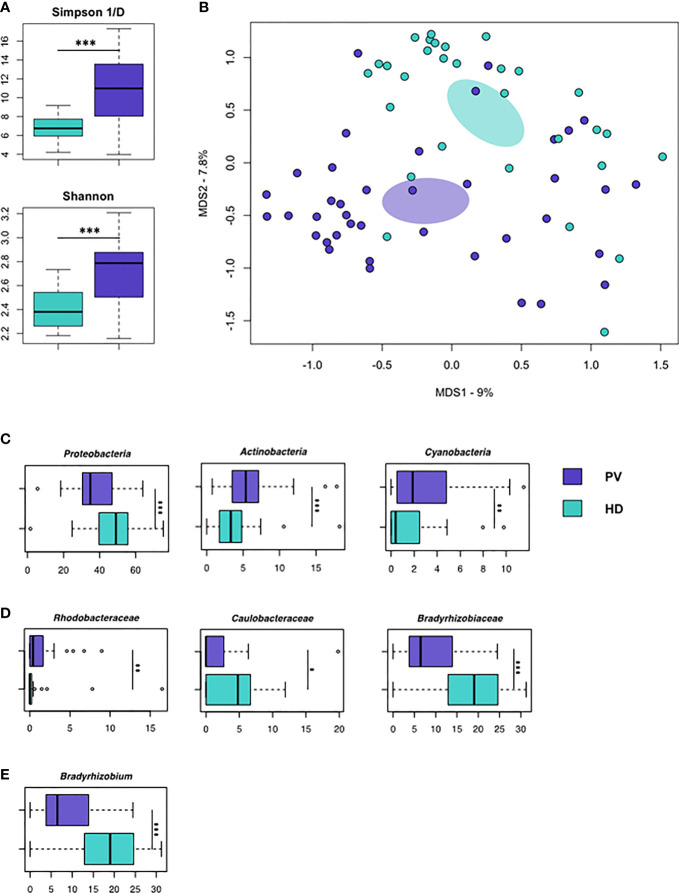
Microbial DNA cargo of isolated EVs in PV patients and HD. **(A)** Alpha diversity estimated according to inverse Simpson (top) and Shannon (bottom) indices. PV patients show significantly higher biodiversity than HD (p < 0.001, Wilcoxon rank-sum test). **(B)** Principal Coordinates Analysis (PCoA) based on Jaccard similarity between the genus-level profiles of EVs from PV patients and HD. Significant segregation between groups was found (p = 0.001, permutation test with pseudo-*F* ratio). **(C–E)** Boxplots showing the relative abundance distribution of phyla, families and genera that were significantly differentially represented between HD and PV patients (*p < 0.05; **p < 0.01; ***p < 0.001; Wilcoxon rank-sum test). Only bacterial taxa with mean relative abundance ≥1% in at least one of the comparison groups are shown.

Isolated EVs were subjected to 16S rRNA gene-based NGS to evaluate whether microbial DNA can be detected and whether its pattern may represent a PV-specific signature. A total of 1,799,864 sequence reads, with an average of 22,220 (± 5,974, SD) reads per sample, were obtained and analyzed. According to the inverse Simpson and Shannon indices, the EVs-associated microbial diversity was higher in PV patients compared to HD (p<0.001, **Figure 2A**). Principal Coordinates Analysis (PCoA) based on Jaccard similarity between the genus-level profiles showed separation between PV patients and HD (p=0.001; **Figure 2B**). Interestingly, taxonomic comparisons revealed numerous differences between the EVs-associated microbial DNA of PV patients and HD. PV patients were depleted in Proteobacteria-related DNA while enriched in that of Actinobacteria and Cyanobacteria, compared to HD (p<0.001; p<0.01; **Figure 2C**). At the family level, the most discriminating taxa (with mean relative abundance ≥1% in at least one of the study groups) were *Rhodobacteraceae* (p<0.01), whose proportions were greater in PV patients, and *Caulobacteraceae* (p<0.05) and *Bradyrhizobiaceae* (p<0.001), which were underrepresented in PV patients (**Figure 2D**). Genus-level data showed depletion of *Bradyrhizobium* in PV patients (p<0.001; **Figure 2E**).

### Characterization of Fecal Microbiome

Fecal samples from PV patients and HD were also profiled by 16S rRNA gene-based NGS. A total of 2,296,462 sequence reads, with an average of 33,772 (± 11,228, SD) reads per sample, were obtained and analyzed. The comparison of the microbiome profiles at the phylum, family and genus level did not reveal any significant difference between PV patients and HD, demonstrating an overall eubiotic intestinal microbial ecosystem in PV (**Figures S4A, B**).

## Discussion

Prior reports showed that serum microparticles originating from platelets, erythrocytes, granulocytes, and endothelial cells of PV patients are elevated compared with healthy controls (32). In addition, it has been described that patients with MPNs that had the JAK2V617F mutation had significantly higher plasma concentrations of tissue factor–positive microparticles and erythrocyte microparticles (33). Here we aimed to identify a signature of PV by analyzing the phenotype and microbial DNA cargo of circulating EVs.

First, we observed that PV is associated with an abnormal profile of circulating EVs of megakaryocyte and platelet origin. Since EV production by megakaryocytes is based on a constitutive mechanism, but only activated platelets can produce CD62P+ EVs (34), our findings further confirm the aberrant megakaryopoiesis and platelet activation, which have been previously described in MPN, including PV patients (1, 35–37). However, this pattern of circulating MK-/PLT-EVs in PV mirrors the profile previously described for MF and ET patients (19), suggesting that abnormal MK-/PLT-EV frequency is common in MPNs, irrespective of disease and mutation status (35, 36).

Secondly, despite our analyses could not discriminate whether EVs were of bacterial or human origin, we found that PV patients had an increased proportion of LPS-associated EVs as compared to HD. These findings suggest not only an increased intestinal permeability in PV but also that, as a cargo of microbial factors such as LPS, EVs could be instrumental for blood microbial components/microbes to mediate their impact on the host inflammatory state and the subsequent activation of the innate immune responses (38). Therefore, we can speculate that in PV the increased proportion of LPS-associated EVs might represent a stimulus for the immune system by boosting key cells such as monocytes/macrophages, dendritic and T cells, thereby stimulating the release of inflammatory/fibrogenic cytokines and contributing to the maintenance of chronic inflammation. However, further investigations are necessary to explore this suggestion.

Finally, PV was associated with a microbial DNA signature of isolated EVs with higher diversity and distinct microbial composition than the healthy counterparts, including greater amounts of Rhodobacteraceae DNA and reduced proportions of DNA related to Caulobacteraceae and Bradyrhizobium. These microorganisms are well known components of soil and aqueous microbiomes and possibly represent a proxy of the personal microbiome exposome, defined as the peculiar pattern of environmental microorganisms we are exposed to.

Of note, it should be underlined that, though in our cohort most PV patients were under treatment at the time of the study and few of them were out of treatment, we were able to demonstrate an abnormal microbial DNA layout when circulating EVs were analyzed. At variance, no significant differences were observed between patients and controls when the gut microbiome was investigated, suggesting that circulating EVs in PV, irrespective of cytotoxic therapy, are biomarker of the disease.

In conclusion, PV is associated with circulating EVs harboring abnormal phenotype and dysbiosis signature with a potential role in the (inflammatory) pathogenesis of the disease. Even though further studies, possibly integrating other-omics approaches on a larger cohort, are warranted to validate our findings and delve into taxonomic, these data might be also of interest in the development of novel personalized therapeutic approaches targeting the microenvironment of PV.

## Data Availability Statement

The data presented in the study are deposited in the National Center for Biotechnology Information Sequence Read Archive (NCBI SRA) repository, accession number PRJNA737425.

## Ethics Statement

The studies involving human participants were reviewed and approved by the Comitato Etico Indipendente di Area Vasta Emilia Centro della Regione Emilia Romagna-IRCCS Azienda Ospedaliero Universitaria di Bologna. The patients/participants provided their written informed consent to participate in this study.

## Author Contributions

FP, MaC and LC designed the study. FP, GA and NV provided clinical information and blood/fecal samples from PV patients. MaB, FR, GCo phenotypically characterized and isolated EVs. FF and EB analysed EV number/size. VP and GCe performed electron microscopy analysis. MoB performed EV/feces NGS study. MaB, MoB, ST, FP and LC wrote the manuscript. MaC, PT and MiC reviewed the manuscript. All authors contributed to interpretation of the data, read and approved the final manuscript.

## Funding

This work was funded by AIL Bologna.

## Conflict of Interest

The authors declare that the research was conducted in the absence of any commercial or financial relationships that could be construed as a potential conflict of interest.

## Publisher’s Note

All claims expressed in this article are solely those of the authors and do not necessarily represent those of their affiliated organizations, or those of the publisher, the editors and the reviewers. Any product that may be evaluated in this article, or claim that may be made by its manufacturer, is not guaranteed or endorsed by the publisher.
