# Supplementary material for: An Abnormal Host/Microbiomes Signature of Plasma-Derived Extracellular Vesicles Is Associated to Polycythemia Vera
Source: Front Oncol. 2021 Nov 25;11:715217. doi: 10.3389/fonc.2021.715217 (PMC8657945; doi:10.3389/fonc.2021.715217)
Supplement: Supplementary file 1 [file DataSheet_1.pdf]

Supplementary Material

1 Supplementary Tables

Supplementary Table 1. Clinical and laboratory features of PV patients and HD. Comparisons of quantitative variables between PV pts and HD were carried out by Mann-Whitney test or chi-square test, as appropriate.

	**PV (38 cases)**	**HD (30 cases)**	**P value**
**Age at study, years; median (range)**	63 (26-84)	66 (51-83)	0.31
**Age >60 years, no. (%)**	24 (63%)	19 (63%)	0.98
**Males, no. (%)**	21 (55%)	12 (40%)	0.21
**Hemoglobin, g/dl; median (range)**	17.7 (13.1-21.8)	13.5 (11.1-15.5)	0.15
**Leukocytes, x 10^9/l; median (range)**	8.9 (5.1-21.5)	5.5 (3.9-9)	<0.001
**Platelets, x 10^9/l; median (range)**	370 (160-813)	230 (124-365)	<0.001
**Hematocrit; median (range)**	55 (40.5-67.6)	41.2 (30.3-47.5)	0.008
**Cardiovascular risk factors, no. of patients (%)**			
smoking	10 (26%)	5 (17%)	0.37
hypertension	24 (63%)	18 (60%)	0.79
diabetes	4 (10.5%)	3 (10%)	0.06
dyslipidemia	10 (26%)	18 (60%)	0.005
**Thrombosis, no. of patients (%)**	14 (37%)	0	<0.001
**Low-dose aspirin, no. of patients (%)**	26 (68%)	3 (10%)	<0.001
**Ongoing antibiotic therapy, no. of patients (%)**	4 (11%)	1 (3%)	0.25
**Ongoing probiotic treatment, no. of patients (%)**	5 (13%)	4 (13%)	0.98

Supplementary Table 2. List of monoclonal antibodies and reagents according to EV subtype.

EVs subtype	Identified as	Monoclonal antibodies/reagents
Megakaryocyte-EVs	CD61+/CD62P-	Anti-CD61 (Clone: SZ21; FITC-conjugated; Catalog number IM1758); Anti- CD62P (Clone: CLB-THROMB/6; PE-coniugated; Catalog number IM1759U). All antibodies from Beckman Coulter S.r.l.
Platelet-EVs	CD61+/CD62P+	Anti-CD61 (Clone: SZ21; FITC-conjugated; Catalog number IM1758); Anti- CD62P (Clone: CLB-THROMB/6; PE-coniugated; Catalog number IM1759U). All antibodies from Beckman Coulter S.r.l.
Tetraspanins-positive EVs	CD81+/CD9+/CD63+	Anti-CD81 (Clone: REA513; APC-conjugated; Catalog number 130-119-825) from Miltenyi Biotec; Anti-CD9 (Clone: REA1071; FITC-conjugated; Catalog number 130-118-806) from Miltenyi Biotec; Anti-CD63 (Clone: H5C6; PE-conjugated; Catalog number 130-100-158) from Miltenyi Biotec.
Lipopolysaccharide-positive EVs	LPS+	Anti-Lipopolysaccharide (LPS) (FITC-conjugated; Catalog number LAB526Ge82) from Cloud-Clone Corp.

2 Supplementary Figures

Supplementary Figure 1. Representative dot-plots of MK-/PLT-EVs and LPS-associated EVs. **(A)** Representative dot-plots of MK-EVs (CD61+CD62P-) and PLT-EVs (CD61+CD62P+) of 1 PV patient and 1 HD; **(B)** Representative dot-plots of LPS-associated EVs after isolation from the plasma of 1 HD and 1 PV patient.

Supplementary Figure 2. Phenotype of EVs isolated from the plasma of PV patients and HD. **(A)** Representative dot-plots of tetraspanins expression (CD81, CD9, CD63) of the total EVs after isolation from the plasma of 1 HD and 1 PV pts**; (B, C** and **D)** Tetraspanins expression (CD81, CD9, CD63) of the total EVs after isolation from the plasma of HD (n=5) and PV patients (n=5). Analysis refers to the total isolated EVs. Data are expressed as mean percentage of tetraspanins-positive EVs ± S.E.M.; **(E, F)** Proportion of megakaryocyte-EVs (MK-EVs; CD61+CD62P-) and platelet-EVs (PLT-EVs; CD61+CD62P+) after isolation from the plasma of PV patients (n=28) and HD (n=20). Analysis refers to the big EVs. Data are expressed as percentage of MK-/PLT-EVs and presented as min to max with median (Mann-Whitney test; **$p<0.01$).

Supplementary Figure 3. Nanoparticle tracking analysis and transmission electron microscopy evaluation of isolated EVs. (A) Concentration of EVs after isolation from the plasma of HD (n=30) and PV patients (n=38). Data are expressed as EVs/mL and presented as min to max with median. **(B)** Mean size of the EVs isolated from the plasma of HD (n=30) and PV patients (n=38). Data are expressed as nm and presented as min to max with median (Mann-Whitney test; ***$p<0.001$). **(C)** Representative histograms showing the EVs-size distribution profile (EVs concentration (EVs × 10^9/mL) vs size in nm) of 1 PV patient and 1 HD. **(D)** Representative images of transmission electron microscopy of EVs after isolation from the plasma of 1 PV patient and 1 HD, showing uniformly round-shaped EVs of different size.

Supplementary Figure 4. The gut microbiome of PV patients and of HD. (A) Alpha diversity estimated according to inverse Simpson (top) and Shannon (bottom) indices. **(B)** Principal Coordinates Analysis (PCoA) based on Jaccard similarity between the genus-level microbiota profiles of PV patients and HD.
