# Supplementary figures and images for: An Abnormal Host/Microbiomes Signature of Plasma-Derived Extracellular Vesicles Is Associated to Polycythemia Vera
Source: Front Oncol. 2021 Nov 25;11:715217. doi: 10.3389/fonc.2021.715217 (PMC8657945; doi:10.3389/fonc.2021.715217)

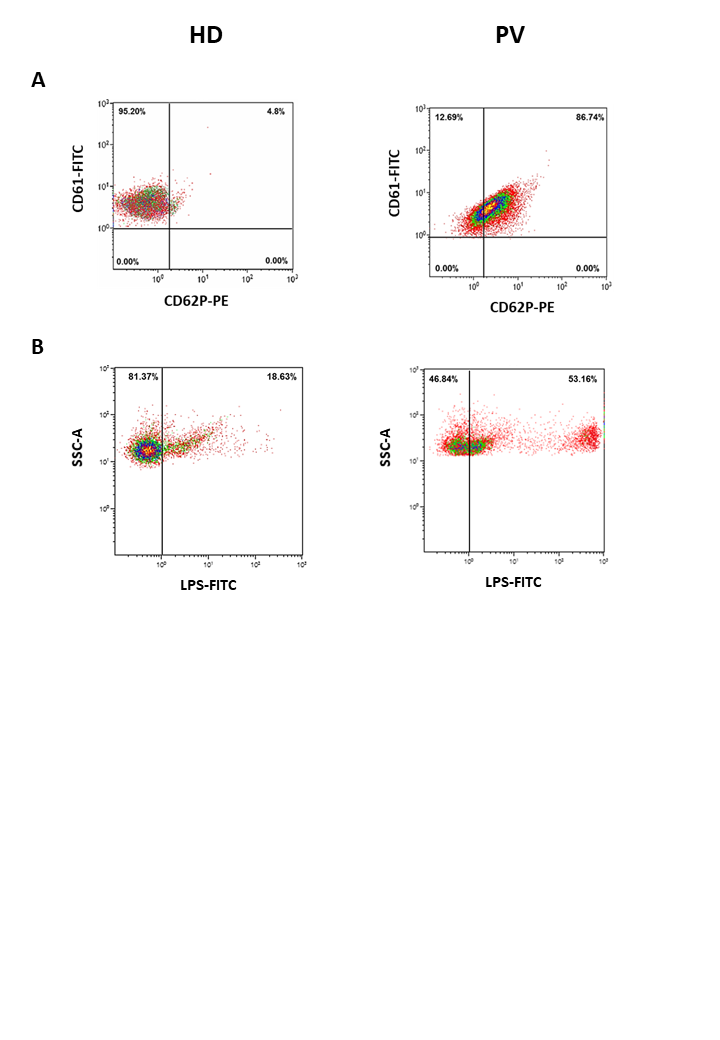

Supplement: Supplementary file 2 [file Image_1.tif]

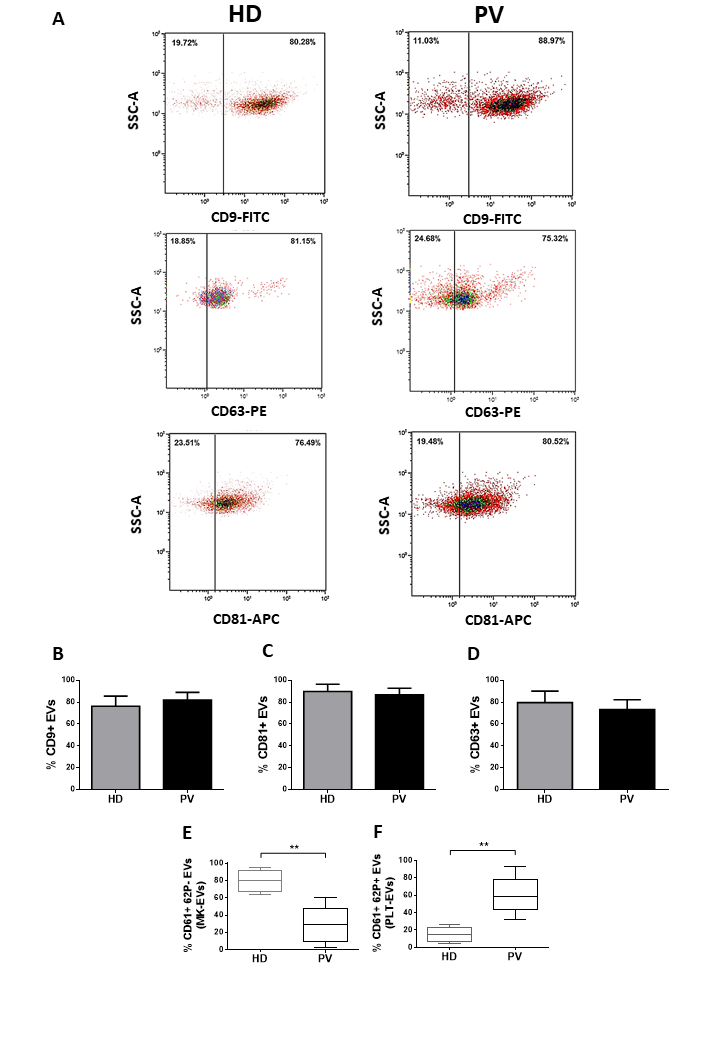

Supplement: Supplementary file 3 [file Image_2.tif]

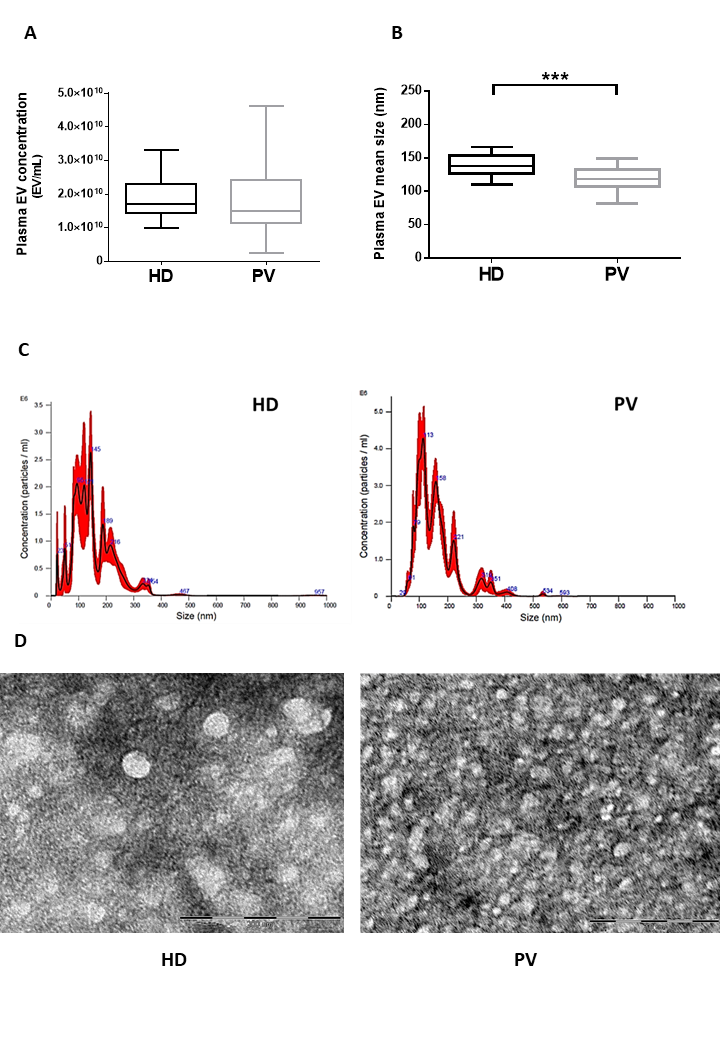

Supplement: Supplementary file 4 [file Image_3.tif]

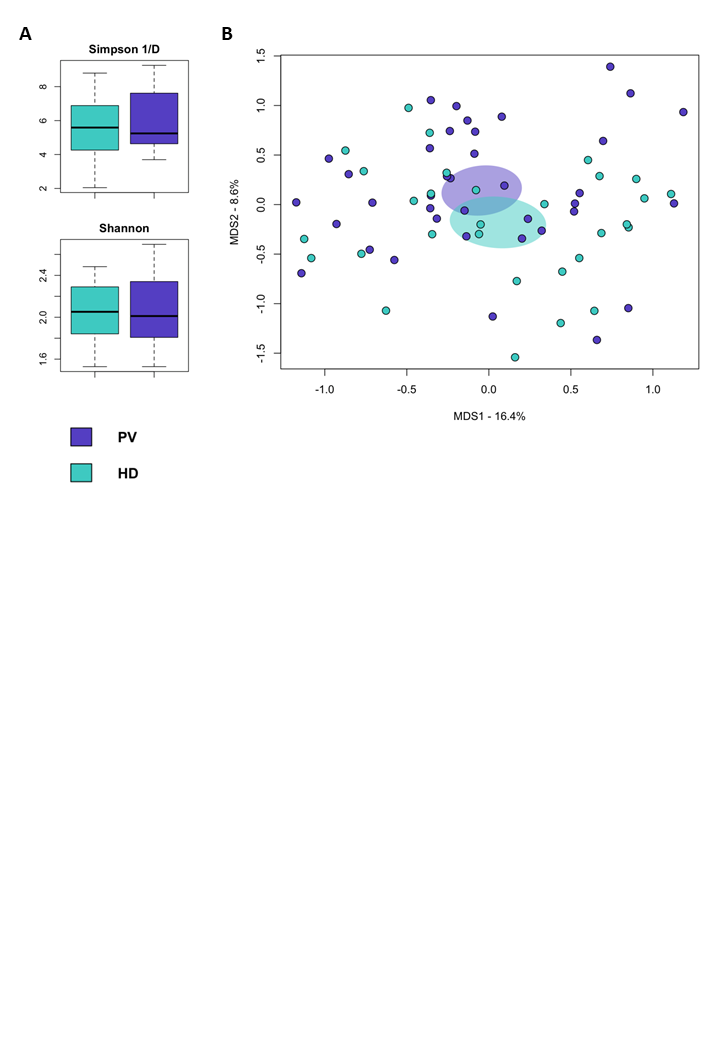

Supplement: Supplementary file 5 [file Image_4.tif]
